# Communication of diagnosis in amyotrophic lateral sclerosis: stratification of patients for the estimation of the individual needs

**DOI:** 10.3389/fpsyg.2015.00745

**Published:** 2015-06-02

**Authors:** Alessia Pizzimenti, Maria Cristina Gori, Emanuela Onesti, Bev John, Maurizio Inghilleri

**Affiliations:** ^1^Department of Neurology and Psychiatry, Sapienza UniversityRome, Italy; ^2^School of Psychology, Early Years and Therapeutic Studies, University of South WalesPontypridd, UK

**Keywords:** ALS, communication, fronto-temporal dementia, diagnosis

Amyotrophic lateral sclerosis (ALS) is a neurodegenerative disease characterized by a progressive loss of the corticospinal tract, brainstem, and spinal neurons, leading to progressive muscle atrophy and weakness, and ultimately to death due to respiratory failure.

The right to be informed is enshrined in the European Oviedo Convention (Council of Europe, 1997). The Awaji diagnostic criteria for ALS are generally used, identifying definite, probable, possible and suspected ALS (de Carvalho et al., 2008). Moreover, the pathway of a correct diagnostic assessment of ALS needs a complex algorithm of knowledge. There are no biochemical markers that allow a definitive diagnosis, and the clinical knowledge of the general practitioner in the early stages is critical in order to direct the patient to the neurologist.

## Psychological assessment

Emotions have not often been studied in ALS. Most existing studies have assessed the psychopathological manifestations involved, essentially depression Ferentinos et al. (2011) and less frequently anxiety. The results have shown that major depressive episodes and anxious episodes are not frequent in ALS patients, although moderate depressive or anxious symptoms are often observed, but less frequently than in other diseases such as multiple sclerosis or Parkinson's disease (Pagnini, 2013).

Patients and their families experience the period of diagnostic assessment for possible ALS with anxiety and distress. When this period is extended, the risk of developing depressive symptoms is greater (Caga et al., 2014). Moreover, a higher prevalence of depression is expected in patients with more severe ALS (Pagnini et al., 2012). In ALS cohorts 7% have minor depression, and 5% have current major depressive disorder (Rabkin et al., 2014). Further, depressive symptoms are significantly related to poorer quality of life (Pizzimenti et al., 2013). A recent study found no correlation between the severity of depression and anxiety and ALS Functional Rating Scale-Revised (ALSFRS-R) score or for disease duration in ALS patients (Chen et al., 2015). However, another investigation of emotional states measured using the Hospital Anxiety and Depression Scale (HADS) showed worsening depression and anxiety scores as ALS progressed (Jones et al., 2014). A recent longitudinal study on “wishing to die” and depression in ALS shows that “wishing to die” is not always expressed in the context of a state of depression and does not necessarily denote psychopathology as such (Rabkin et al., 2014). It is iportant to note that ALS can also be associated not only with psychological problems but also with the impairment of many neuropsychological functions.

## Comorbidity with ftd

ALS and FTD could be two ends of the spectrum of one disease (Taes et al., 2010). Approximately 15% of ALS patients show Fronto-Temporal Dementia (FTD) with TDP43 positive inclusions in cortical neurons, whereas at least 50% of them evidence subtle cognitive and/or behavioral dysfunction (Lillo and Hodges, 2009; Lillo et al., 2014). Patients with clinical evidence for both disorders have ALS-FTD. Furthermore, many patients with ALS show some cognitive or behavioral changes without meeting the criteria for FTD; they are described as ALS with cognitive or behavioral impairment (ALS-Ci/Bi). Patients with FTD can similarly show evidence of mild motor neuron involvement without developing ALS: they are said to have FTD-MND.

A significant delay in the time-course of selective attentive processing and a difficulty in initiating and sustaining attention may be present in ALS-b, which points to the possibility of dysfunction in the frontal neural network that responds to novelty and to abnormal integration of associative functions. This attentional impairment should be taken into account when developing alternative communicative strategies in ALS patients (Mannarelli et al., 2014). Although FTD patients exhibit prominent deficits in emotion perception and social cognition, these domains have received relatively little attention in ALS. Performance on emotion processing tasks may provide a useful clinical tool in identifying patients with early FTD-ALS (Savage et al., 2014). Structural and functional neuroimaging have demonstrated that ALS is associated with abnormalities localized mainly in the frontal lobes, and neuropathological investigations have shown the pathological involvement of the prefrontal cortex (Troakes et al., 2012). In FTD structural brain imaging and neuropsychological data involve the functions of the prefrontal cortex. Damage to this region is associated with deficient performance in theory of mind and in affective decision-making, but the relationship between these two capacities in patients with prefrontal cortex dysfunction is unclear. Further neuropsychological or functional studies are indicated as necessary to improve early identification of patients affected by FTD. Comorbidity with FTD should be considered where ALS is diagnosed, and importantly, also when this diagnosis is communicated to patients.

## When, what, and how to communicate diagnosis

ALS is a deadly disease. The appropriate ways to communicate the diagnosis to patients could be similar to the strategies used in other serious pathologies, such as some variations of cancers with short survival rates.

If ALS is associated with FTD, both diagnoses should be communicated to patients. In this regard the evidence base of good practice in the communication of dementia diagnoses may also be useful for FTD-ALS. For example, in managing newly diagnosed ALS patients, physicians need to consider the following questions: “when, what, and how to communicate the diagnosis?”

### When to communicate the diagnosis?

The communication of the diagnosis is a complex event: it contains “informative,” “prognostic,” and “therapeutic” components. The “informative” component or phase provides and defines factual information regarding the medical condition. The “prognostic” component describes the short and long-term implications for the patient and family. The therapeutic component consists of the proposal of a practical plan for management (Karnieli-Miller et al., 2007). In ALS the time of the communication of the diagnosis will often coincide with the communication of prognosis and therapy. The time taken for the informative component is generally shorter than for the other components, which could be reflective of the discomfort of the physician in delivering bad news.

The difficulties for the medical team in making a diagnosis of ALS, and in the moment of communication of the diagnosis to patients has been reported in a recent study (Schellenberg et al., 2014). Physicians and other healthcare workers should learn and implement effective methods for delivering information on the patient's health status (Marcus and Mott, 2014).

The communication process must consider:

– The creation of a strong therapeutic alliance between the entire multidisciplinary team and the patient. A good alliance is critical to subsequent discussion of treatment options (Back et al., 2009). Only within a relationship of reciprocal confidence and trust can the communication of a disease like ALS be understood and processed by the patient. The psychologist is crucial to the creation of this alliance (Marcus and Mott, 2014).
– The comorbidity with FTD and the stage of disease. If the progression of dementia is fast, the communication of diagnosis should be earlier. The diagnosis of FTD and ALS should be communicated when the patient is still able to understand it and to choose his/her own future strategies to cope with his/her illness in accordance with national laws.
– The comorbidity with depression, taking psychological support into account, using measures of risk of suicide. Suicide risk does not exempt the physician from the communication of the diagnosis at a more opportune time.
– The psychological support to the patient and caregiver before and after communication of diagnosis (Miller et al., 2009).

### What to communicate?

The communication about prognosis and evolution in ALS is complicated by the variability of the clinical course and the possible psychopathological and neurological comorbidities. Moreover, patients with ALS-FTD can have increased difficulties with, or lack cognitive resources for, decision-making. In all forms of dementia, despite its importance and implications, the format, content and process of this communication has received insignificant research attention.

The communication should be patient centered and include:

– A clear description of diagnosis, prognosis and therapeutic choices. A truthful but compassionate communication between physicians and patients is essential for decisions about both disease-directed (curative) and palliative therapies (Rich, 2014).
– Feedback of the patient's understanding about his/her knowledge and acceptance of the disease. This is very important in the case of FTD. The FTD would present impaired anosognosia-related implicit awareness due to a dysfunctional implicit integration of contextual information caused by an abnormal fronto-insular-temporal network (Ibáñez et al., 2013). In FTD, the inhibition, self-monitoring, set-shifting, and mood orientation changes appear to be important skills for awareness of instrumental activities of daily living, while hypo manic mood orientation and a tendency for apathy to be prominent are indications of reduced behavioral awareness (Amanzio et al., 2013).
– The monitoring of the awareness of the patient at the time. Some elderly patients can refuse to receive information about their illness and can abdicate to the paternalistic model of medicine, while other patients tend to search all possible avenues for information. In a survey in Germany 28% of patients and 23% of caregivers used (alternative) resources to find information related to their symptoms before seeing a doctor, mainly from the internet. After the medical visit, although two-thirds were satisfied with the means-disclosure of diagnosis, 88% of patients and 85% of caregivers searched for additional information, most often online and by reading brochures from patients' associations (Abdulla et al., 2014).
– The presence of relatives or caregivers, only if it is accepted by the patient.

### How to communicate the diagnosis?

Communication of ALS diagnosis may be psychologically traumatic, and patients may need to cope with this by using defense mechanisms as well as individual resources such as resilience. Generally physicians may be unprepared to break bad news, mostly because of a lack of training in emotional management (Pagnini et al., 2012). The relationship between the communication of a negative diagnosis and the response of the patients will depend to a great extent on the manner in which the message is transmitted, but to our knowledge these aspects have been poorly studied in motor neuron diseases.

The method of communicating a diagnosis of ALS should include:

– The application of techniques of counseling, including a comfortable space, adequate time for processing information, respect for the patient and his/her reactions, acceptance of the patient's reactions.
– The possibility of countertransference: the individual health professional should pay particular attention to his or her personal emotions, such as feelings about death.
– Existing evidence based knowledge of methods of communication, for example the Six-Step Protocol for Delivering Bad News used in cancer care (Kaplan, 2010). This includes: setting up the interview, assessing the patient's perception, obtaining the patient's invitation, giving knowledge and information to the patient, addressing the patient's emotions with empathic responses, strategy, and summary.
– The use of simple words that can be understood easily by the patient. The use of euphemisms, vague words, and not naming the disease could be a defense mechanism of the physician (Karnieli-Miller et al., 2007).

In conclusion, communicating a diagnosis of ALS should be done in a way that empowers patients and caregivers in order to improve the disease management and decisions about treatment options. To be effective, it should be individualized, by taking into account several features including:

- Stage of the disease at the moment of the diagnosis and possible cognitive impairment;.
- Personal characteristics (including personality traits, personal values, possible psychopathological complications like depressive phenomena and mood liability);.
- Social and cultural environment of the patient and his/her family, in order to harmonize the communicative process with the values and cultural aspects of the family.

Being part of the medical team and involved from the very beginning in all phases of the healing process, by supporting physicians in their role, the psychologist can prove to be helpful in enhancing it indirectly, as well as directly supporting patients and caregivers.

### Conflict of interest statement

The authors declare that the research was conducted in the absence of any commercial or financial relationships that could be construed as a potential conflict of interest.
